# Hemostatic safety profile of estetrol vs. ethinylestradiol in oral contraceptives: a systematic review and meta-analysis

**DOI:** 10.61622/rbgo/2026rbgo27

**Published:** 2026-07-17

**Authors:** Maria Julia Lemos, Jacqueline Maia Ferraz, Laura Fonseca Queiroz, Alice França Diniz, Celene Maria Longo da Silva, Priscila Luiza dos Santos, Patricia Myriam Antunes de Oliveira Gomide, Andrea Mora De Marco Novellino

**Affiliations:** 1 Centro Universitário Itaperuna Department of Medicine Itaperuna RJ Brazil Department of Medicine, Centro Universitário Itaperuna, Itaperuna, RJ, Brazil; 2 Universidade Federal da Bahia Department of Medicine Vitória da Conquista BA Brazil Department of Medicine, Universidade Federal da Bahia, Vitória da Conquista, BA, Brazil; 3 Santa Casa de Misericórdia de Belo Horizonte Department of Obstetrics and Gynecology BH Brazil Department of Obstetrics and Gynecology, Santa Casa de Misericórdia de Belo Horizonte, BH, Brazil; 4 Escola Superior de Ciências da Saúde Department of Medicine Brasília DF Brazil Department of Medicine, Escola Superior de Ciências da Saúde, Brasília, DF, Brazil; 5 Universidade Federal de Pelotas Department of Medicine Pelotas RS Brazil Department of Medicine, Universidade Federal de Pelotas, Pelotas, RS, Brazil; 6 Faculdade Santa Marcelina Department of Medicine São Paulo SP Brazil Department of Medicine, Faculdade Santa Marcelina, São Paulo, SP, Brazil; 7 Unimed - BH Department of Obstetrics and Gynecology Belo Horizonte MG Brazil Department of Obstetrics and Gynecology, Unimed - BH, Belo Horizonte, MG, Brazil; 8 Universidade Federal do Paraná Department of Obstetrics and Gynecology Curitiba PR Brazil Department of Obstetrics and Gynecology, Universidade Federal do Paraná, Curitiba, PR, Brazil

**Keywords:** Estetrol, Coagulation, D-dimer, Thrombin, Hemostatic

## Abstract

**Objective:**

To evaluate the hemostatic profile of estetrol/drospirenone (E4/DRSP) compared with ethinylestradiol/drospirenone (EE/DRSP) in adult women.

**Methods:**

A systematic review and meta-analysis of randomized controlled trials (RCTs) was performed according to PRISMA guidelines. Eligible studies compared E4/DRSP with EE/DRSP in adult women and assessed coagulation, anticoagulation, or fibrinolytic markers. Three RCTs (n=183) met inclusion criteria. Data were extracted independently, and pooled mean differences (MD) were calculated using random-effects models.

**Results:**

Compared to EE/DRSP, E4/DRSP was associated with higher protein S activity (MD = 21.23; 95% CI: 11.83 to 30.64; p<0.00001) and lower levels of fibrinogen (MD = −24.17; 95% CI: -39.15 to -9.19; p=0.002), plasminogen (MD = −27.28; 95% CI: -27.28 to – 22.89; p<0.00001), and SHBG (MD = −183.38; 95% CI: -183.38; 95% CI: -210.93 to -155.84; p<0.00001). No significant differences were observed for D-dimer and antithrombin. Protein C activity was higher in the EE group, though this was an isolated finding with limited interpretability.

**Conclusion:**

E4/DRSP demonstrates a more favorable hemostatic profile than EE/DRSP, potentially reducing thrombotic risk. Despite the limited number of RCTs, consistency across markers supports E4 as a safer estrogenic component for contraceptive formulations, with implications for women at increased risk of venous thromboembolism.

**PROSPERO registry**: #CRD420251074961

## Introduction

Combined oral contraceptives (COCs) is one of the most widely used birth control methods worldwide, offering high efficacy and additional non-contraceptive benefits, such as hyperandrogenic treatment and dysfunctional uterine bleeding.^(1)^ However, their use is associated with an increased risk of venous thromboembolism (VTE), primarily due to estrogen-induced changes in hepatic synthesis of coagulation and fibrinolysis proteins.^(2-4)^

Ethinylestradiol (EE) is a synthetic derivative of 17β-estradiol, and it is the most widely used estrogen in combined oral contraceptives (COCs). It exerts a strong hepatic effect, stimulating the synthesis of procoagulant proteins such as fibrinogen and sex hormone-binding globulin (SHBG), while concurrently downregulating natural anticoagulants, including protein S.^(5)^ While Estetrol (E4) is a fetal estrogen with an unique tissue-selective activity, and it has been emerging as a potential alternative due to its reduced hepatic impact and favorable pharmacologic profile.^(6)^

Although individual trials have reported favorable hemostatic changes with estetrol-based contraceptives compared to those containing ethinylestradiol, evidence remains limited to a small number of randomized studies with modest sample sizes. These trials suggest that estetrol combined with drospirenone (DRSP) may induce fewer procoagulant shifts and preserve natural anticoagulant activity when compared to EE/DRSP. However, until now, no meta-analysis has systematically synthesized these data to quantify the magnitude and consistency of estetrol's impact on the coagulation cascade.

Given the widespread use of COCs and the clinical relevance of minimizing thrombotic risk, we conducted a systematic review and meta-analysis to evaluate the effect of E4/DRSP versus EE/DRSP on coagulation and fibrinolysis parameters. We used the PICO framework: (P) adult women using combined oral contraceptives; (I) estetrol combined with drospirenone (E4/DRSP); (C) ethinylestradiol combined with drospirenone (EE/DRSP); and (O) changes in hemostatic and fibrinolytic biomarkers, including protein S activity, protein C activity, antithrombin, fibrinogen, plasminogen, D-dimer, and sex hormone-binding globulin (SHBG).

## Methods

### Protocol

This systematic review and meta-analysis was performed and reported in accordance with the Cochrane Collaboration Handbook for Systematic Review of Interventions and the Preferred Reporting Items for Systematic Reviews and Meta-Analysis (PRISMA) Statement guidelines.^(7,8)^

### Eligibility criteria

Inclusion in this meta-analysis was restricted to studies that met all the following eligibility criteria: (1) randomized controlled trials; (2) adult women (>18 years old) out of menopause; (3) primary outcome evaluating coagulation markers; (4) follow up with no restriction. We excluded studies with (1) women with contraindications for the use of contraceptive steroids; (2) studies without a comparison group with ethinylestradiol/drospirenone; (3) studies that do not evaluate coagulation markers as an outcome; (4) studies without a clear definition of the methods used to assess outcome. No restrictions were applied regarding the language of publication or the duration of the follow-up period. The decision of limiting our meta-analysis to RCTs is primarily driven by the need to maximize the internal validity and reliability of your findings, thereby strengthening the evidence base for clinical recommendations or understanding causal relationships.

### Search strategy and Data extraction

We systematically searched PubMed, Embase, and the Cochrane Central Register of Controlled Trials from inception to June 2025 using the following search terms: ‘estetrol’, ‘coagulation’, ‘d-dimer’, ‘thrombin’ and ‘hemostatic’. Full search strategy for each database can be assessed through Supplementary Material – Table 1S. The references from all included studies, previous systematic reviews and meta-analyses were also searched manually for any additional studies. Two authors (M.J.L and J.M.F.) independently extracted the data following predefined search criteria and quality assessment. No automated data extraction tools were used. Disagreements were resolved through consensus among the authors. The prospective meta-analysis protocol was registered on PROSPERO on June 16th, 2025, under protocol #CRD420251074961.

### Data synthesis

Data synthesis was conducted using a structured and predefined approach. Two reviewers independently extracted all relevant data, including study design, sample size, intervention and comparator characteristics, and outcomes of interest (hemostatic and fibrinolytic biomarkers). Extracted data were cross-checked for accuracy before analysis. Summary quantitative data were then pooled for meta-analysis using appropriate statistical models as described in the statistical analysis section.

### Endpoints and subgroup analysis

The primary outcome assessed was the alterations of coagulation markers associating the use of combined oral contraceptive with E4/DRSP compared with the use of EE/DRSP. We assessed coagulation markers evaluating anticoagulant factors as antithrombin, protein C activity and protein S activity, procoagulant factors as fibrinogen, fibrinolytic factors as plasminogen, and markers for ongoing coagulation as D-dimer. Three studies reported antithrombin, protein C activity (PCa), protein S activity (PSa) and SHBG.^(9-11)^ Two studies reported D-dimer, fibrinogen and plasminogen.^(9,11)^

### Quality assessment

We evaluated the risk of bias in randomized studies using version 2 of the Cochrane Risk of Bias assessment tool (RoB 2).^(12)^ All assessments were independently performed by two reviewers (M.J.L. and J.M.F.), and disagreements were resolved through a consensus after discussing reasons for discrepancy.

### Statistical analysis

Effect measures included standardized mean differences (SMDs) for continuous outcomes. In cases where continuous data were reported as medians with interquartile ranges, the standard deviation was approximated using validated statistical approaches recommended by the Cochrane Handbook, assuming normal data distribution. Heterogeneity was examined with the Cochran Q test, I^2^ statistic, and was considered significant if the p-value was less than 0.10, the I^2^ statistic exceeded 25%. For this study, we used DerSimonian and Laird random-effects model. To assess the robustness of the results, we performed leave-one-out sensitivity analyses. All statistical analyses were conducted using Review Manager version 5.4 (The Cochrane Collaboration, Copenhagen, Denmark).

## Results

### Study selection and baseline characteristics

**Figure 1:**
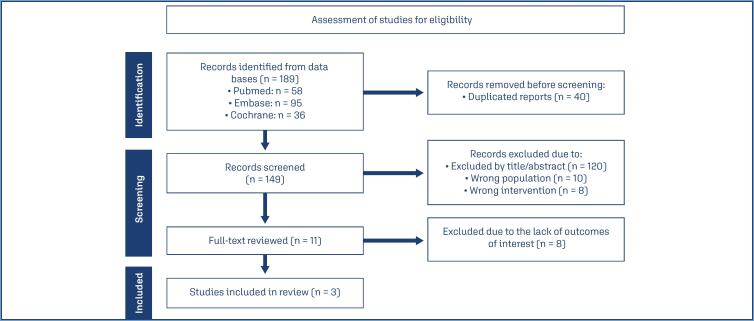
PRISMA flow diagram of study screening and selection

The initial search yielded 189 results, of which 40 articles were duplicate records, and 120 articles were deemed unrelated based on title or abstract review and were excluded. After removal of duplicate records and ineligible studies, 11 remained and were carefully reviewed based on inclusion criteria. After evaluating inclusion and exclusion criteria, 3 randomized controlled trials were included in this systematic review and meta-analysis, comprising a total of 183 patients (Figure 1).^(9-11)^ Of the total study population, 97 participants (53%) received E4/DRSP, and 86 participants (47%) received EE/DRSP. Two studies evaluated 15mg E4 with 3mg DRSP.^(9,11)^ In all studies, the control group received 20 µg EE combined with DRSP, whereas one study evaluated 10mg E4 combined with 3mg DRSP as intervention.^(10)^ Oral treatment was started on the first day of menstruation in all studies.^(9-11)^ Study measurements were assessed by blood sample analysis. Douxfils et al. (2020)^(9)^ collected samples pre-treatment, in the middle of cycle 3 and cycle 6. One study collected one blood sample pre-treatment, at the final of cycle 1 and cycle 3, and post-treatment cycle.^(10)^ Another study collected blood samples pre-treatment, at the final of cycle 1 and cycle 3.^(11)^ All blood was collected in a citrate tube.

The three RCT were conducted between 2017 and 2024. Sample sizes ranged from 32 to 82 women per study. Participants were young reproductive-age women, with mean ages ranging from 23.8 to 34.9 years and mean BMI between 20.8 and 22.9 kg/m^2^. Treatment duration varied from 120 to 180 days. While all studies assessed core hemostatic parameters such as antithrombin, protein C activity, protein S activity, and SHBG, only two studies reported fibrinogen, plasminogen, and D-dimer. Detailed study characteristics are summarized in Table 1.

**Table 1:** Baseline characteristics of included studies

Author	Year	Study design	Patients (n) Intervention / control	Age (years) Mean ± SD (total)	BMI# (kg/m2) Mean ± SD (Intervention/Control)	Follow up	Outcome measures
Douxfils et al.^(9)^	2020	RCT^	38/31	26.2±7.25	22.85±2.925	180 days	AT@, D-dimer, Fibrinogen, Plasminogen, PCa$, PSa&, SHBG*
Kluft et al.^(10)^	2017	RCT^	15/17	23.8±3.75	22.92±2.925	120 days	AT@, PCa$, PSa&, SHBG*
Kobayashi et al.^(11)^	2024	RCT^	44/38	34.9±7.61	20.76±2.440	120 days	AT@, D-dimer, Fibrinogen, Plasminogen, PCa$, PSa&, SHBG*

@ AT: Antithrombin Activity

# BMI: Body Mass Index

$ PCa: Protein C Activity

& PSa: Protein S Activity;

^ RCT: Randomized Controlled Trial;

* SHBG: Sex Hormones Binding Globulin.

### Pooled analysis of included studies

**Figure 2:**
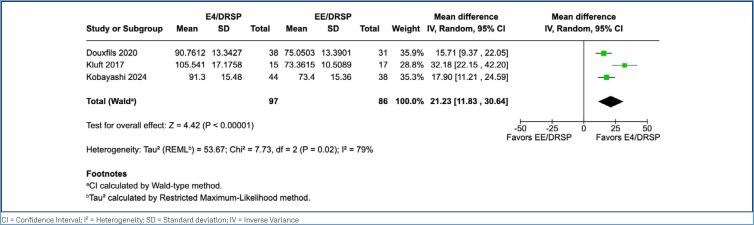
Protein S activity increased to a greater extent with E4/DRSP compared to EE/DRSP, suggesting a more favorable effect on the endogenous anticoagulant pathway

**Figure 3:**
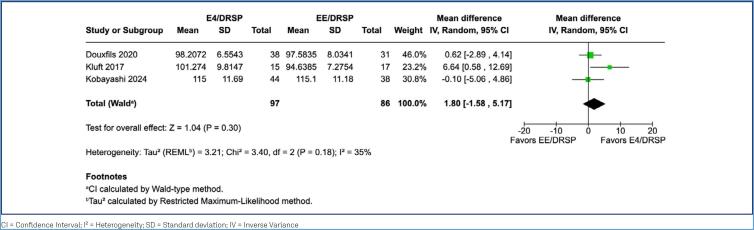
Although not statistically significant, antithrombin activity tended to be higher in the E4/DRSP group compared to EE/DRSP

**Figure 4:**
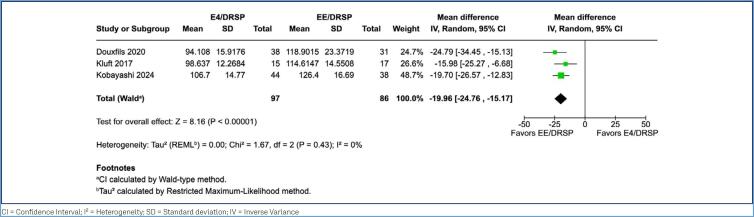
Protein C activity was significantly greater in the EE/DRSP group compared to E4/DRSP

When evaluating individual anticoagulant parameters, E4/DRSP consistently showed a more favorable profile compared to EE/DRSP. When comparing protein S activity, we observed a lower hemostatic impact of E4/DRSP when compared to EE/DRSP (MD = 21.23; 95% CI 11.83 to 30.64; p<0.00001; I^2^=79%) (Figure 2). Trends favoring E4/DRSP were observed for antithrombin activity, although these did not reach statistical significance (MD = 1.80; 95% CI -1.58 to 5.17; p=0.30; I^2^=35%) (Figure 3). A significantly greater increase in protein C activity was observed in the EE/DRSP group compared to E4/DRSP (MD = −19.96; 95% CI −24.76 to -15.17; p<0.00001; I^2^=0%) (Figure 4), suggesting a more favorable effect on this specific anticoagulant marker.

**Figure 5:**
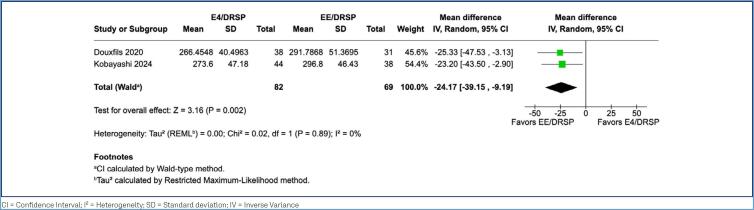
EE/DRSP has a statistically significant impact on fibrinogen levels compared to E4/DRSP, contributing to a more procoagulant environment

Among the procoagulant factors, only fibrinogen levels could be evaluated, as key parameters such as prothrombin, factors VII, VIII, Xa, prothrombin time, and activated partial thromboplastin time were not consistently reported across studies. Moreover, fibrinogen levels were available in only two studies,^(9,11)^ reported fibrinogen activity rather than serum concentration. Despite the limited number of studies reporting fibrinogen levels, the available evidence consistently indicates higher levels with EE/DRSP compared to E4/DRSP, which contributes for a more procoagulant environment in the EE/DRSP group (MD = -24.17; 95% CI -39.15 to -9.19; p=0.002; I^2^=0%) (Figure 5), nevertheless not statistically significant.

**Figure 6:**
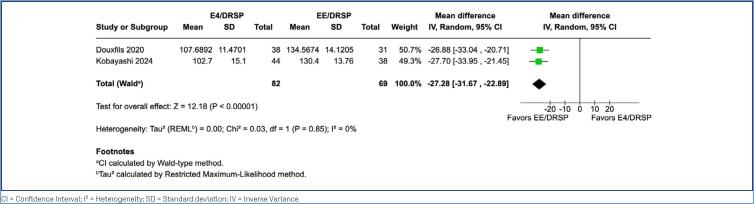
An increased plasminogen was observed in the EE/DRSP group, indicating a markedly more procoagulant fibrinolytic profile when compared to E4/DRSP

**Figure 7:**
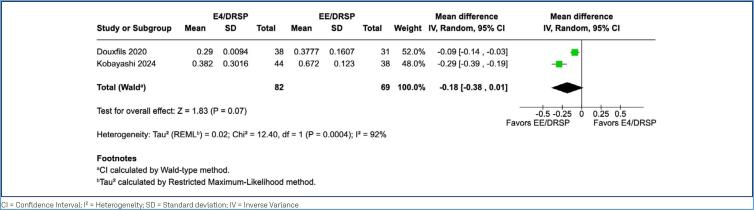
No statistically differences were observed between groups when evaluating D-dimer. However, there is a trend to the EE/DRSP group, suggesting a potentially more procoagulant profile when compared to E4/DRSP

Regarding fibrinolytic factors such as plasminogen, D-dimer, tissue plasminogen activator (tPA), soluble fibrin monomer complex (SFMC), and plasminogen activator inhibitor-1 (PAI-1), only plasminogen and D-dimer could be assessed due to limited data across studies. Moreover, only two studies reported these parameters. Nevertheless, plasminogen levels appear to increase with EE/DRSP compared to E4/DRSP, suggesting a shift toward a more procoagulant fibrinolytic profile (MD = -27.28; 95% CI -31.67 to -22.89; p<0.00001; I^2^=0%) (Figure 6), while D-dimer presented no statistical difference between groups (MD = -0.18; 95% CI -0.38 to 0.01; p=0.07; I^2^=92%) (Figure 7).

**Figure 8:**
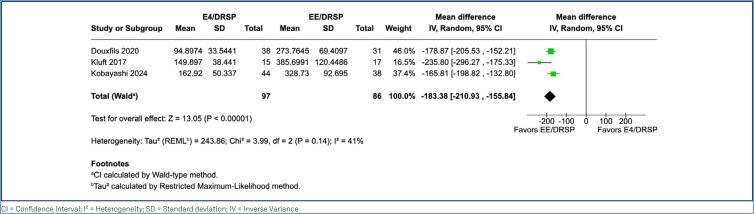
SHBG levels were significantly lower in users of E4/DRSP compared to EE-containing contraceptives, supporting a more favorable hormonal impact on hepatic metabolism with a consequent favorable hemostatic profile

Beyond the evaluation of direct hemostatic markers, sex hormonal binding globulin (SHBG) was included as a biological marker reflecting hepatic estrogenic activity.^(13)^ Our analysis revealed significantly lower SHBG levels in users of E4/DRSP versus EE-containing regimens (MD = -183.38; 95% CI -210.93 to -155.84; p<0.00001; I^2^ = 41%) (Figure 8), supporting the observed trend toward a more favorable hemostatic profile.

Taken together, the findings suggest that E4/DRSP exerts a milder and potentially safer impact on the coagulation cascade compared to EE/DRSP. This was reflected in several hemostatic markers, including higher levels of anticoagulant proteins, such as protein S, lower levels of procoagulant indicators as fibrinogen, and trends toward more favorable fibrinolytic profiles. However, the magnitude and consistency of these effects varied across biomarkers, likely due to differences in study design, analytical methods, and reporting standards. Despite these limitations, the overall pattern consistently favors E4/DRSP as having a reduced prothrombotic potential.

### Sensitivity analysis

To address the observed heterogeneity in some outcomes, a leave-one-out sensitivity analysis was conducted by sequentially excluding each study to assess the influence of individual studies on the overall estimate. The results remained consistent as the exclusion of any single study did not materially alter the alterations of hemostatic markers.

### Quality assessment

The Risk of Bias 2 (Rob-2) was used for quality assessment.^(12)^ No study was considered at critical risk of bias as described in Figure 9. The GRADE evidence assessment applied to these outcomes is presented in Table 2, detailing the ratings for each domain and the overall certainty of the evidence. The overall certainty of the evidence was rated as high.

**Table 2:** Summary of findings and certainty of evidence rated according to the GRADE methodology. Outcomes were classified as high, moderate, low, or very low certainty.

Certainty assessment							№ of patients		Effect		Certainty	Importance
№ of studies	Study design	Risk of bias	Inconsistency	Indirectness	Imprecision	Other considerations	E4 + DRSP	EE + DRSP	Relative(95% CI)	Absolute(95% CI)		
Protein C Activity resistance (follow-up: mean 140 days)												
3	randomised trials	not serious	not serious	not serious	not serious	none	183	183	-	0(24.76 lower to 15.17 lower)	⨁⨁⨁⨁ High	IMPORTANT
Antithrombin activity (follow-up: mean 120 days)												
3	randomised trials	not serious	not serious	not serious	not serious	none	151	183	-	0(1.58 lower to 5.17 higher)	⨁⨁⨁⨁ High	IMPORTANT
Plasminogen												
2	randomised trials	not serious	not serious	not serious	not serious	none	151	151	-	0(31.67 lower to 22.89 lower)	⨁⨁⨁⨁ High	IMPORTANT
D-dimer												
2	randomised trials	not serious	not serious	not serious	not serious	none	151	151	-	0(0.38 lower to 0.01 higher)	⨁⨁⨁⨁ High	IMPORTANT
Fibrinogen (follow-up: mean 120 days)												
2	randomised trials	not serious	not serious	not serious	not serious	none	151	151	-	0(39.15 lower to 9.19 lower)	⨁⨁⨁⨁ High	IMPORTANT
SHBG Level (follow-up: mean 140 days)												
3	randomised trials	not serious	not serious	not serious	not serious	none	183	183	-	0(210.93 lower to 155.84 lower)	⨁⨁⨁⨁ High	IMPORTANT

## Discussion

In this systematic review and meta-analysis of three randomized trials, we assessed the effects of E4/DRSP and EE/DRSP on coagulation and fibrinolysis markers. The main finding was that EE/DRSP exerted a significantly greater impact on the hemostatic system, as evidenced by pooled mean differences in key parameters, including reductions in protein S activity, and elevations in fibrinogen and plasminogen levels when compared to E4/DRSP. Although protein C activity was significantly lower in the E4/DRSP group, this isolated marker does not fully reflect the broader hemostatic profile or other clinical outcomes. Considering that the remaining markers consistently indicated a more favorable hemostatic effect in the E4/DRSP group, the overall profile supports its potential safety advantage.

Beyond its favorable effects on coagulation markers, estetrol-based combined oral contraceptives have demonstrated high acceptability and user satisfaction, with reported improvements in premenstrual symptoms and favorable weight control outcomes.^(13)^ These benefits are thought to stem from estetrol's unique pharmacological profile, acting as an agonist in target tissues such as bone, uterus, and brain, while exerting minimal hepatic stimulation. This selective activity supports its classification as the first Native Estrogen with Selective action in Tissues (NEST).^(14)^

While the overall direction of the results was consistent, certain differences among the studies may have contributed to the observed heterogeneity in some outcomes, such as protein S activity. One potential explanation lies in the diversity of populations included. Two studies were conducted among healthy reproductive-age women.^(9,10)^ Whereas one focused on women with endometriosis, a condition that may independently influence hemostatic balance through inflammatory and hormonal pathways.^(11)^ Additionally, slight variations in dosage regimens, treatment duration, and laboratory assays may have affected absolute values, although the relative changes remained aligned.

The studies were also conducted in distinct geographical regions, including Europe and Asia, which may contribute both to the generalizability of the findings and to subtle differences in baseline risk factors.^(9-11)^ Such diversity is a strength in terms of external validity, yet it may introduce methodological heterogeneity that complicates direct comparisons across studies. Differences in sampling intervals, laboratory techniques, and definitions of hemostatic endpoints also represent potential sources of variation.

Furthermore, the biomarkers analyzed in this study vary in clinical relevance and sensitivity to hormonal changes. For example, SHBG is not a coagulation factor per se but is often used as an alternate marker for estrogenic hepatic stimulation.^(15)^

Similarly, D-dimer reflects fibrin degradation and overall fibrinolytic activity, however, its levels are highly influenced by factors such as age, systemic inflammation, and assay variability, making it an unreliable biomarker for consistent evaluation in this context.^(16)^ While its reduction with E4 did not reach statistical significance, the directionality observed across trials suggests a potential effect that may become clearer in larger studies with more statistical power.

Our findings consistently suggest reduced hemostatic activation with E4-based contraceptives across multiple biomarkers. This is clinically relevant given that ethinylestradiol-induced changes in coagulation parameters are a major contributor to the elevated risk of venous thromboembolism in oral contraceptive users and represent a common reason why many women opt against their use.^(17)^ Although our analysis did not include direct data on VTE events, the markers assessed, particularly fibrinogen, protein S, protein C and SHBG, are mechanistically linked to thrombotic risk.^(18,19)^

The heterogeneity observed in the protein S activity analysis (I^2^ = 86%) may reflect variability in patient populations and measurement methods. However, other outcomes such as fibrinogen (I^2^ = 0%) and plasminogen (I^2^ = 0%) demonstrated remarkable consistency, suggesting that the overall findings are robust and not driven by outliers or single studies. Importantly, the fact that these outcomes are based on randomized comparisons increases confidence in the internal validity of the results, despite the limited number of included studies.

As with any research, this study has its limitations. Firstly, the number of included studies was small, limiting the possibility of performing subgroup analyses or assessing publication bias. Secondly, the studies were not designed or powered to assess clinical outcomes such as thromboembolic events, and thus our findings should be interpreted as indirect evidence of improved safety based on hemostatic markers. Thirdly, while the included studies had similar designs, methodological differences may have influenced results, and the absence of data from longer follow-up periods prevents us from extrapolating these findings to long-term use.

## Conclusion

In this systematic review and meta-analysis, we provided the first synthesized evidence suggesting that combined oral contraceptives containing estetrol and drospirenone (E4/DRSP) are associated with an overall more favorable hemostatic profile compared to ethinylestradiol-based formulations. These findings may have implications for contraceptive counseling and the development of new oral formulations of hormonal therapies, particularly for women at increased risk of thrombosis. Future studies should focus on large-scale trials incorporating clinical endpoints to hemostatic biomarkers, and extended follow-up periods to confirm whether the favorable hemostatic parameter profile observed here translates into reduced thrombotic risk in real-world use.

## Data Availability

The research data are described in the article presented.
