# Supplementary material for: Hemostatic safety profile of estetrol vs. ethinylestradiol in oral contraceptives: a systematic review and meta-analysis
Source: Rev Bras Ginecol Obstet. 2026 Jul 17;48:e-rbgo27. doi: 10.61622/rbgo/2026rbgo27 (PMC13399374; doi:10.61622/rbgo/2026rbgo27)
Supplement: Supplementary Material [file 1806-9339-rbgo-48-e-rbgo27-Suppl01.pdf]

Supplementary Material

Table 1S. Full search strategy

**Pubmed**	(“Estetrol”[Mesh] OR estetrol OR E4[tiab]) AND (“Hemostasis”[Mesh] OR hemostasis[tiab] OR “Blood Coagulation”[Mesh] OR “Fibrin Fibrinogen Degradation Products”[Mesh] OR “Thrombin”[Mesh] OR thrombin[tiab] OR “blood coagulation”[tiab] OR “D-dimer”[tiab] OR prothrombin[tiab] OR fibrinolysis[tiab] OR fibrinogen[tiab] OR “coagulation factor”[tiab] OR hemostatic[tiab])
**Embase**	(‘estetrol’/exp OR estetrol OR ‘e4’/exp OR e4) AND (‘hemostasis’/exp OR hemostasis OR ‘thrombin’/exp OR thrombin OR ‘blood coagulation’/exp OR ‘blood coagulation’ OR ‘d-dimer’/exp OR ‘d-dimer’ OR ‘prothrombin’/exp OR prothrombin OR ‘fibrinolysis’/exp OR fibrinolysis OR ‘fibrinogen’/exp OR fibrinogen OR ‘coagulation factor’/exp OR ‘coagulation factor’ OR hemostatic)
**Cochrane**	(estetrol OR E4) AND (hemostasis OR thrombin OR “blood coagulation” OR “d-dimer” OR prothrombin OR fibrinolysis OR fibrinogen OR “coagulation factor” OR hemostatic)
